# Acute air embolism caused by autotransfusion during percutaneous atrial septal defect closure: A case report

**DOI:** 10.1002/ccr3.5654

**Published:** 2022-03-27

**Authors:** Yu‐Qi Liao, Meng‐Qiu Zhang

**Affiliations:** ^1^ Department of Anesthesiology The Third People's Hospital of Chengdu Chengdu China; ^2^ Department of Anesthesiology West China Hospital Sichuan University Chengdu China

**Keywords:** air embolism, atrial septal defect, autologous blood transfusion, case report

## Abstract

Atrial septal defect is a common congenital heart disease in adults and it is often asymptomatic. Percutaneous device closure is gaining popularity, but percutaneous repair of atrial septal defect leading to left atrial rupture and subsequent autotransfusion under high pressure leading to air embolism has not been reported yet.

## 1 INTRODUCTION

Percutaneous atrial septal defect (ASD) closure is currently a routine surgical procedure performed by a cardiac surgeon or cardiologist. Potential complications of this procedure include cardiac perforations, device malposition or embolization, residual shunts, vascular trauma, thrombus formation, atrioventricular valve regurgitation, cardiac arrhythmias, infectious endocarditis, and even sudden death.^1^ Intraoperative autotransfusion plays a definite role in the management of cardiovascular crisis as exemplified in our patient described below. However, a case of intraoperative acute air embolism caused by autotransfusion during percutaneous ASD closure has not yet been reported. Occasionally, we have experienced both heart rupture and air embolism at the same time and our experiences of successfully dealing with it may help other anesthesiologists.

## 2 CASE PRESENTATION

A 30‐year‐old woman was admitted for congenital ASD with a left to right shunt with New York Heart Association (NYHA) Class I and sinus rhythm. She had a cold 3 months ago, and the diagnosis of secundum type ASD was established in the course of medical treatment. The anatomy of the ASD was evaluated by transthoracic echocardiography (TTE) before surgery. The prior TTE demonstrates that it was a single defect with maximum diameter 36 mm in all views. The position relationships of ASD with surrounding structures such as superior vena cava, coronary sinus, right upper pulmonary vein, and atrioventricular valves were also assessed. The distances from the margins of the defect to those surrounding structures were all over 5 mm. Right ventricular chamber was slightly enlarged (18 × 38 mm). Additional congenital heart defects such as partial anomalous pulmonary venous return and pulmonary arterial hypertension were excluded. It was just a usual secundum type ASD which was eligible for percutaneous device closure. She was in a good general condition without cough, fever, heart failure, or constitutional symptoms before surgery. Her vital signs were stable. She also had no special past history or family history.

Elective percutaneous closure of the single ASD was guided by intraoperatively transesophageal echocardiography (TEE). After successful anesthesia induction, left radial artery catheterization (Allen's test was negative) and right central venous catheterization were established. After percutaneous puncture of the femoral vein and the rail track was constructed, a 10F long delivery sheath was advancing over the super smooth guide wire into the left atrium. As the delivery sheath passed through the ASD successfully under the guidance of TEE and entered the left atrium, the left atrial appendage was ruptured by careless mistake due to excessive forward movement. The heart rate increased immediately, and blood pressure started to decrease. An immediate thoracotomy was performed to repair the left atrial appendage gap immediately, while the perfusionists were preparing for emergency cardiopulmonary bypass. The anesthesiologist called for help as the circulation collapsed. First‐aid approaches were taken such as applying blood salvage techniques, accelerating infusion, and administrating vasoactive drugs to restore and maintain circulation stabilization.

**FIGURE 1:**
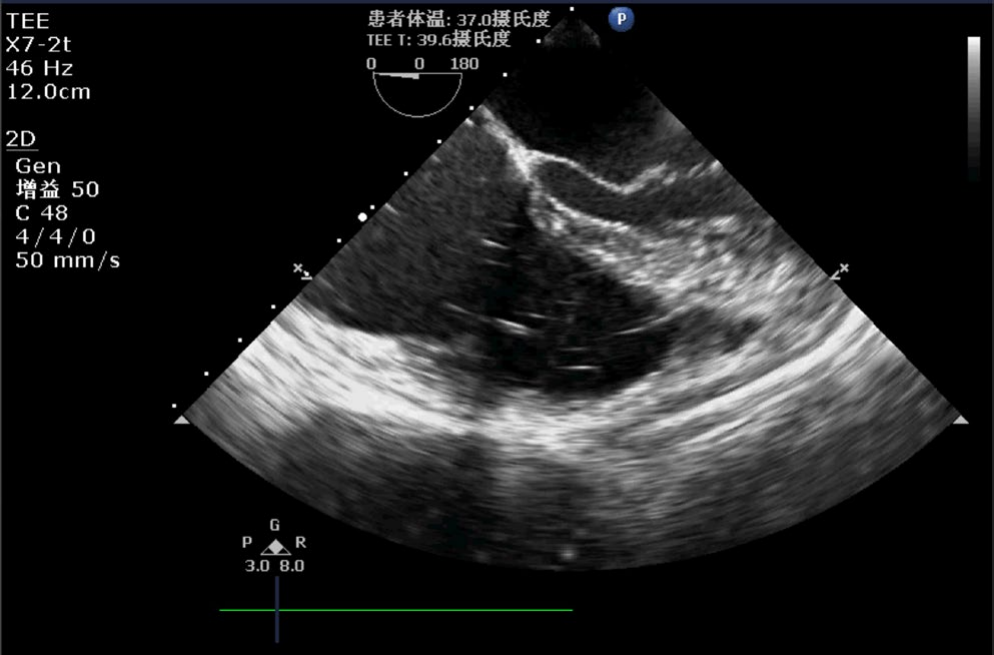
Transesophageal echocardiography demonstrated air within heart chambers

The surgeon sawed the chest and found the tear immediately. The blood was sucked into the cell saver reservoir continuously at the same time. Approximately 10 min later, the reservoir was filled with salvaged blood. A total of 600 ml autologous blood was pressurized and rapidly transfused through the central vein after washing, centrifugation, and filtration. Blood pressure fluctuated between (60–100)/(40–65) mmHg. The tear was sewn up shortly without cardiopulmonary bypass. Approximately 15 min later, the monitor suddenly alarmed again, indicating that the pressure of the end‐tidal carbon dioxide (P_ET_CO_2_) waveform decreased and that the measured value was only 9 mmHg. The blood pressure dropped dramatically to 52/31 mmHg, and the heart rate increased to 131 bpm. The integrity of the respiratory circuit and fluid access were immediately checked. Autologous blood had already been transfused, and central venous access was filled with air, with a small amount of residual blood hanging sporadically in the tube wall. At the same time, the TEE image showed scattered bubbles in the right atrium and the right ventricle outflow tract, with significant enlargement of the right heart, which indirectly reflected the signs of pulmonary hypertension. Thereafter, the diagnosis of pulmonary gas embolism was established (Figure 1). However, no bubbles were observed in the left ventricular chamber. These findings hint that the residual air of the reinfusion blood bag had been squeezed into the vena cava by the indirect‐pressure interlayer‐inflated transfusion bag.

The central venous access was clamped at once, the pressure bag was deflated, and the body position was tilted to the right side with the Trendelenberg position. A 50 ml syringe was used to try to extract gas from the central vein. Vasoactive drugs were actively administered to stabilize circulation, and respiratory parameters were regulated to maintain oxygenation during the operation. Pulse oxygen saturation plunged to 75% after 3 min. Then, 100% oxygen ventilation and cerebral protection were performed. Repeated intravenous injections of 20 μg of norepinephrine and 100 μg of epinephrine were given intermittently and the infusion of inotropes to stabilize the circulation.

Approximately 20 min after rescue, the vital signs gradually stabilized. Arterial blood gas analyses were performed, and electrolyte acid‐base balance was restored. Glucocorticoid was used to alleviate inflammation. After a short time‐out discussion, our team decided to terminate the operation. The patient was transferred to the cardiothoracic intensive care unit. On the first day after surgery, transient myocardial markers were elevated and inflammatory exudation was obvious in the lung. As shown by the head computed tomography scan, no cerebral infarction was observed. After 2 days of respiratory therapy and circulatory support, she was extubated and transferred to a general ward. She was discharged on the eighth day after surgery. Six months later, she underwent uneventful transcatheter closure of ASD performance in our hospital and had a good prognosis.

## 3 DISCUSSION

Atrial septal defect is one of the most common congenital heart diseases in adults, and it occurs two to three times more often in women than in men.^2^, ^3^ The feasibility of percutaneous closure of ASD was first reported in 1976 by Terry et al.^4^ This procedure is becoming increasingly popular due to the short learning curve, cosmetic benefits, reduced pain, and reduced hospital stay. Nevertheless, complications with occasional deaths have been reported. Other more common complications include cardiac perforations, device malposition or embolization, residual shunts, vascular trauma, thrombus formation, cardiac arrhythmias, and infectious endocarditis.^1^ The technique‐related cardiac perforations occasionally occurred during catheterization. The feeling of resistance in the surgeon's hands is critically important. However, the antero‐superior atrial wall and adjacent aorta are uniquely vulnerable. The surgeon even did not feel any sense of a breakthrough when a heart was torn by the sheath. In our case, the left atrial appendage was ruptured by the sheath. An intraoperative echocardiographer (IE), generally an anesthesiologist, plays the role of the “eye” of the surgeon. The IE should alert the surgeon in real time about where the sheath tip is.

The first known documented procedure of autotransfusion was performed in 1818 by Dr. James Blundell.^5^ Venous air embolism (VAE) is a relatively rare but potentially fatal event compared with other complications of autotransfusion such as blood loss if not properly connected, blood contamination resulting in infection, hemolysis due to suction or degradation, and thrombocytopenia.^6^ It may occur in a variety of procedures and surgeries but is most often associated with iatrogenic complications of central line catheter insertion.^7^ There were warnings regarding autotransfusion usage. For example, the first item of the warning list of the SURETRANS™ autotransfusion system for orthopedics is “DUE TO THE POTENTIAL FOR AIR EMBOLISM, DO NOT PRESSURE REINFUSE WHILE USING THE SURETRANS™ AUTOTRANSFUSION TRANSFER BAG.” However, the practice of externally pressurizing reinfusion blood bags is still common and widespread.^8^ Consequently, other additional solutions to minimize the risk of fatal VAE appear to be desirable and necessary. Strategies such as avoiding pressuring the cell saver reinfusion blood bag, insertion of an air bubble detector with an audible alarm in the infusion line to the patient, and routine use of a transfer bag (remove air and disconnect and exchange the transfer bag) may help to prevent VAE during autotransfusion.^8^ A proposal of removal of all air from the autotransfusion bag and lines before administering the salvaged blood to the patient was strongly recommended by Marek et al.^9^ Moreover, according to our experience, what will greatly minimize the risk of VAE is to handle the cell saver by one special person. TEE is the most sensitive tool for the diagnosis of VAE and for documenting the exact passage through cardiac chambers. Preparation for procedures at high risk for VAE and TEE warrants serious consideration which could detect as little as 0.02 ml/kg of air administered by bolus injection.^10^ In our case, the TEE was used from the beginning to the end.

In addition to our emergency management of VAE, a more comprehensive summary of the management principles of VAE has been proposed by Marek et al,^11^ such as the prevention of further air entrainment, aspiration of air from the right atrium, hemodynamic support, and hyperbaric oxygen therapy.

Catastrophic acute VAE caused by autotransfusion during percutaneous ASD closure has not been reported. In this case, the occlusion device itself can produce air embolism during the procedure. In addition, the ruptured heart itself can lead to air embolism too. The effect of air embolism depends upon both the rate and the volume of air introduced into the circulation.^12^ The lethal volume has been described as between 200 and 300 ml, or 3–5 ml/kg.^11^ It first manifests as a drop in P_ET_CO_2_ in intubated general anesthesia. When paradoxical air embolism occurs, it may lead to extreme sympathetic excitation and a transient increase in blood pressure. Fortunately, this dangerous event did not occur in our case. There was no severe systemic circulatory embolism or organ lesion, such as cerebral infarction. In general, iatrogenic gas embolism is associated with high long‐term mortality and morbidity.^13^ Bessereau et al^13^ highlighted the crucial role of hyperbaric oxygen therapy because they found that a time to hyperbaric oxygen therapy of 7 h or less reduced the risk of mortality and neurological sequelae, while the type and source of gas embolism had little impact on outcome.

## 4 CONCLUSION

Transcatheter closure of ASD is gaining popularity. But the procedure‐related complications should not be neglected. Though severe air embolism is rare, it can be life‐threatening once it occurs. Therefore, it is very important that the surgical team work together and hold the line on safety. Once VAE is considered, The IE should scan immediately and make a diagnosis, and imaging results should also be preserved. Effective circulatory and respiratory support should be maintained, and hyperbaric oxygen therapy should be given accordingly.

## CONFLICT OF INTEREST

The authors declare that they have no competing interests.

## AUTHOR CONTRIBUTIONS

Liao YQ contributed to the clinical conduct of the study, data collection, and writing of the manuscript. Zhang MQ contributed to analysis and interpretation of the collected data, writing, preparation of accompanying figures and material, and revision of the manuscript.

## ETHICAL APPROVAL

This study was conducted in accordance with the 1964 Declaration of Helsinki and its later amendments.

## CONSENT

Written informed consent was obtained from the patient for publication of this case report and any accompanying images.

## Data Availability

All data generated during this study can be accessed through direct communication with the corresponding author and the agreement of all research team members.
